# Lack of Genetic Interaction between *Tbx20* and *Tbx3* in Early Mouse Heart Development

**DOI:** 10.1371/journal.pone.0070149

**Published:** 2013-07-25

**Authors:** Svetlana Gavrilov, Richard P. Harvey, Virginia E. Papaioannou

**Affiliations:** 1 Department of Genetics and Development, Columbia University Medical Center, New York, New York, United States of America; 2 Developmental and Stem Cell Biology Laboratory, Victor Chang Cardiac Research Institute, Darlinghurst, New South Wales, Australia; 3 St. Vincent's Hospital Clinical School, University of New South Wales, Kensington, New South Wales, Australia; Institut Jacques Monod, France

## Abstract

Members of the T-box family of transcription factors are important regulators orchestrating the complex regionalization of the developing mammalian heart. Individual mutations in *Tbx20* and *Tbx3* cause distinct congenital heart abnormalities in the mouse: *Tbx20* mutations result in failure of heart looping, developmental arrest and lack of chamber differentiation, while hearts of *Tbx3* mutants progress further, loop normally but show atrioventricular convergence and outflow tract defects. The two genes have overlapping areas of expression in the atrioventricular canal and outflow tract of the heart but their potential genetic interaction has not been previously investigated. In this study we produced compound mutants to investigate potential genetic interactions at the earliest stages of heart development. We find that *Tbx20; Tbx3* double heterozygous mice are viable and fertile with no apparent abnormalities, while double homozygous mutants are embryonic lethal by midgestation. Double homozygous mutant embryos display abnormal cardiac morphogenesis, lack of heart looping, expression patterns of cardiac genes and time of death that are indistinguishable from *Tbx20* homozygous mutants. Prior to death, the double homozygotes show an overall developmental delay similar to *Tbx3* homozygous mutants. Thus the effects of *Tbx20* are epistatic to *Tbx3* in the heart but *Tbx3* is epistatic to *Tbx20* with respect to developmental delay.

## Introduction

The four-chambered mammalian heart develops from a simple linear heart tube by polar elongation, myocardial differentiation and morphogenesis. This complex regionalization of the developing heart is orchestrated by multiple signaling modules and transcriptional circuits. Members of the T-box family of transcription factors are particularly important regulators of myocardial proliferation and patterning. T-box family members *Tbx1, Tbx2, Tbx3, Tbx5, Tbx18* and *Tbx20* are expressed in the heart and regulate various aspects of embryonic heart development. The importance of unperturbed T-box gene function is highlighted by mutations in mice and human, which are associated with congenital heart disease [1–3]. Patients heterozygous for mutations in the coding region of *TBX20* suffer from diverse cardiac defects, including ventricular septal defects, aberrant valvulogenesis, tetralogy of Fallot and cardiomyopathy [4–7]. Mutations in *TBX3* result in the ulnar-mammary syndrome in which a subset of patients has ventricular septal defects [8] or conduction defects [9].

In the mouse, *Tbx20* is expressed throughout the first heart field, in a subset of second heart field progenitors and at later stages in the endocardium and derived mesenchyme of the atrioventricular and outflow tract (OFT) cushions and the atrioventricular septum [10–13]. Following heart looping, *Tbx20* expression decreases in the chamber myocardium compared with atrioventricular and cardiac outflow regions [11,12]. Several studies show that mice lacking *Tbx20* die early during development and display short, severely underdeveloped heart tubes that fail to undergo looping [3,13–15]. Failure in the deployment or recruitment of the second heart field is likely a contributing factor to this phenotype, since elongation of the heart tube is primarily achieved by recruitment of cardiac progenitors originating from the second heart field rather than by proliferation. The expression pattern of *Tbx20* is compatible with both cell-autonomous and non-cell-autonomous defects of this process. Chamber myocardial genes are not activated in *Tbx20* deficient hearts while *Tbx2*, which is normally restricted to the atrioventricular canal (AVC) and OFT, is ectopically expressed throughout the cardiac crescent and linear heart tube [3,13–15]. *Tbx20* heterozygous mice survive postnatally but show dilated cardiomyopathy, which phenocopies at least a subset of human *TBX20* mutant defects [3].

In the mouse heart, *Tbx3* expression is first detected in the inflow tract at the onset of heart looping. Its expression delineates the developing cardiac conduction system, endocardial cushions in the AVC, and the mesenchyme of the OFT [16,17]. *Tbx3* enables development of the cardiac conduction system by restricting cell division and repressing the chamber-specific gene expression program. *Tbx3* deficient embryos are developmentally retarded and die at midgestation apparently due to yolk sac deficiencies although *Tbx3* mutant hearts are also abnormal [18,19]. Tbx3 deficiency results in variable heart defects including increased cell division in the atrioventricular canal, incomplete ventricular septation, double outlet right ventricle, and delayed aortic arch formation. Homozygous mutants also show a failure of atrioventricular convergence, the process by which the inflow region is displaced dorsally to the ventricular segment during heart looping [19,20].

Thus, when individually mutated, *Tbx20* and *Tbx3* cause distinct congenital heart abnormalities, but their overlapping expression in the AVC and outflow tract in the developing heart raises the question of whether they act independently or through transcriptional regulation of common target genes. *Tbx20* expression appears ectopically in the apex of the ventricular septum of *Tbx3* mutant hearts at E12.5 [10] and *Tbx3* expression is markedly down regulated at E9.5 in the AVC of embryos with a conditional deletion of *Tbx20* [21]. However, the potential genetic interaction between these two genes has not been investigated with respect to phenotypic effects. In this study, we investigated the phenotype of *Tbx20*; *Tbx3* double homozygous mutants to explore possible genetic interaction between these two genes based on their overlapping expression in the AVC and OFT. With respect to heart development, the double homozygous phenotype was indistinguishable from that of the *Tbx20* single homozygous mutant in that heart looping and development was arrested early in development. In addition, a general developmental delay characteristic of *Tbx3* homozygous mutants was apparent in the double homozygous mutants, apparently independent of the cardiac phenotype.

## Materials and Methods

### Mice

All experiments were carried out in strict accordance with the recommendations in the Guide for the Care and Use of Laboratory Animals of the National Institutes of Health under protocols approved by the Columbia University Institutional Animal Care and Use Committee (protocol No. AC-AAAD3302). Mutant alleles were maintained on a mixed genetic background derived from 129, C57BL/6Tac and ICR mice. Mice heterozygous for a null mutation of the *Tbx20* locus, *Tbx20* ^*lacZ*^ [3], hereafter designated as *Tbx20* ^*-*^, or a null mutation of the *Tbx3* locus, *Tbx3* ^*tm1Pa*^ [18], hereafter designated as *Tbx3* ^*-*^, were intercrossed to produce double heterozygotes, which were intercrossed to generate double homozygotes as well as other genetic combinations. Embryos and weanling mice were genotyped for *Tbx20* and *Tbx3* by PCR from lysates of yolk sac or tail tip, respectively. The mutant allele of *Tbx20* was genotyped with primers: Primer 1 (LoxP F2): 5’-GACTGGAGAGGCCATCAAAA-3’ and Primer 2 (LacZR2): 5’-GTTTTCCCAGTCACGACGTT- 3’; and the wild type allele was genotyped with primers: T20 wt sense: 5”-CCCAAGGAGAAGGAGGCAGCAGAGAAC-3’ and T20 wt antisense: 5’- CGCAAGTATAAAATGGGGGTTCCTGACC-3’. PCR conditions were 3 minutes at 94^o^C, 30 cycles (30 seconds at 94^o^C, 30 seconds at 61^o^C, 60 seconds at 72^o^C), and 5 minutes at 72^o^C. The primers and PCR conditions for *Tbx3* were as previously described [18].

### Embryo collection and *in situ* hybridization

For timed pregnancies, females were placed with males overnight and checked the following morning for the presence of vaginal plugs. Noon on the day of the plug was designated E0.5. Embryos between E8.5 and E9.25 were dissected out of the uteri and their extra-embryonic membranes in phosphate buffered saline (PBS) with 0.2% bovine serum albumin and were fixed in 4% paraformaldehyde in PBS overnight. Standard procedures were used for whole mount *in situ* hybridization [22].

### Statistics

Statistical analyses were performed using the Chi-square distribution, Mann Whitney U test and notched box plot analysis (Excel, Graph Pad Prism and R http://www.wessa.net/rwasp_notchedbox1.wasp).

## Results

### Generation of *Tbx20* ^*-/-*^ *; Tbx3* ^*-/-*^ double homozygous mutants

Mice heterozygous for *Tbx20* ^*-*^ or *Tbx3* ^*-*^ were intercrossed to generate compound heterozygotes, *Tbx20* ^*+/-*^ *; Tbx3* ^*+/-*^, which were viable and fertile and present at the expected Mendelian frequency at weaning (Χ^2^=4.08; p>0.10; Table 1). No apparent abnormalities were observed. Compound heterozygotes were intercrossed and embryos of all possible genotypes were recovered at midgestation at the expected Mendelian frequency (Χ^2^ = 14.38; p>0.05; Table 1).

**Table 1:** Number of offspring of each genotype from intercrosses of *Tbx20* and *Tbx3* heterozygotes, recovered at weaning, and from crosses of mice double heterozygotes for both genes, recovered at midgestation.

Cross	Genotypes										Total
	*Tbx20*	+/+	+/-	+/+	+/-	-/-	-/-	+/+	+/-	-/-	
	*Tbx3*	+/+	+/+	+/-	+/-	+/+	+/-	-/-	-/-	-/-	
Tbx20^+/-^ x Tbx3^+/-^		28	23	18	16						85*
*Tbx20* ^*+/-*^; Tbx3^+/-^ x Tbx20^+/-^; *Tbx3* ^*+/-*^		11	15	13	30	12	21	6	6	12	126**

* Recovered and genotyped at weaning.

** Recovered and genotyped E8.5 - E9.25.

### Embryonic lethality and abnormal cardiac morphogenesis in *Tbx20; Tbx3* compound mutant embryos

**Figure 1:**
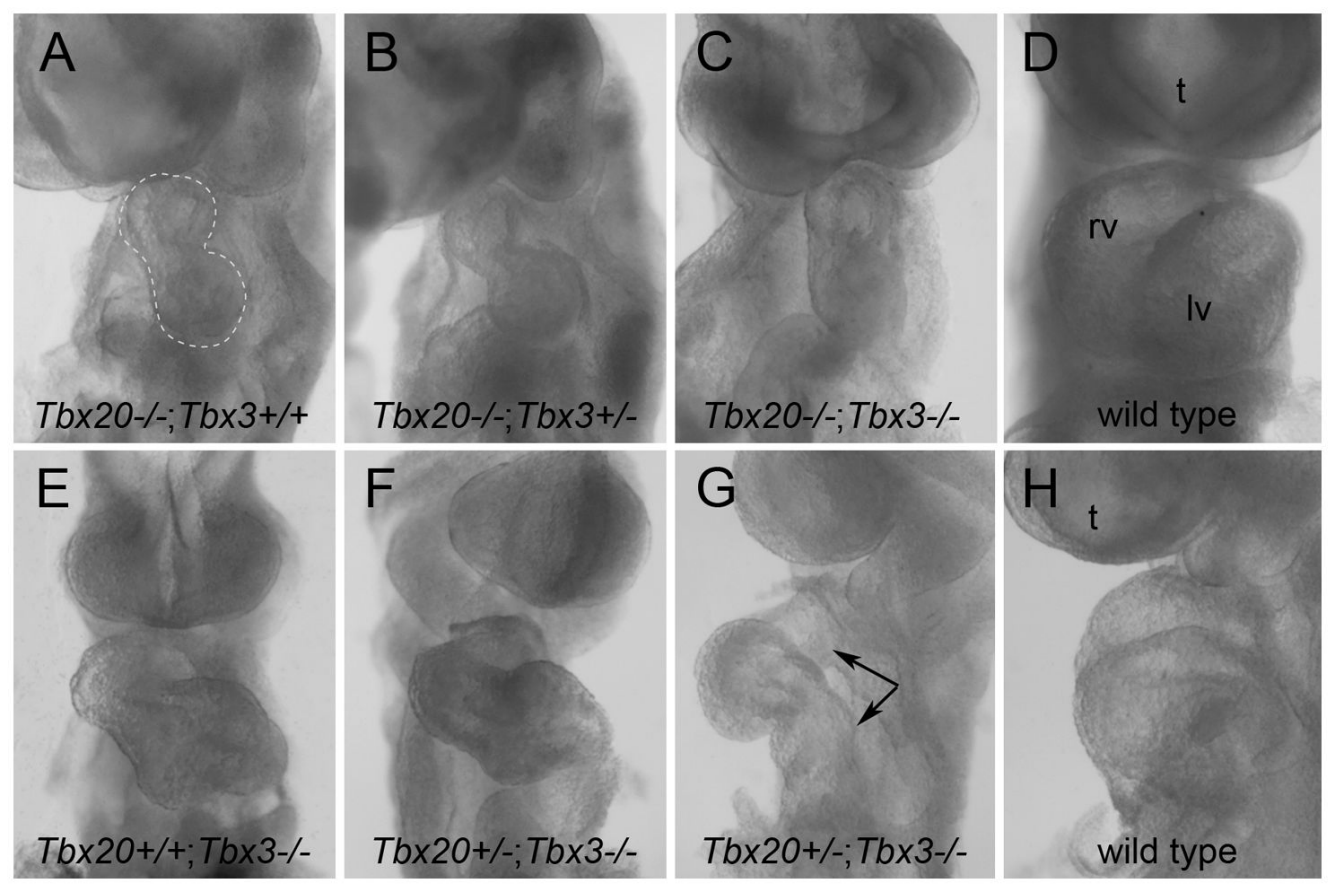
*Tbx20* ^*-/-*^ *; Tbx3* ^*-/-*^ double homozygotes show heart defects similar to *Tbx20^-/-^* embryos. Whole mount ventral (A–F) and left side (G, H) views of the heart (E9.25; 15-20 somite embryos) of *Tbx20; Tbx3* single, compound and double mutants compared with wild type (D, H). All *Tbx20* ^*-/-*^ embryos have an unlooped, hourglass-shaped heart (A-C, outlined in A), whereas *Tbx3* ^*-/-*^ embryos either wild type or heterozygous for *Tbx20* (E, F, G) have normally looped hearts but show atrioventricular convergence defects (G, arrows) compared with wild type (H). lv, left ventricle; rv, right ventricle; t, telencephalon.

At E8.5 and E9.25, embryos (n=210) from heterozygous matings were scored for heart looping and other morphological and developmental features. Of these, 126 embryos were generated by double heterozygous intercrosses while 84 embryos were generated by crossing Tbx20^+/-^ *;* Tbx3^+/-^ double heterozygotes to single heterozygotes. All embryos homozygous mutant for *Tbx20*, including Tbx20^-/-^; Tbx3^+/+^ (n=27), Tbx20^-/-^ *;* Tbx3^+/-^ (n=29), as well as double homozygous mutant embryos (n=14), were morphologically indistinguishable from one another at any given developmental stage: Starting at the heart looping stage, heart tube formation was retarded, hearts were small, and heart looping failed, resulting in two vertically oriented chamber-like swellings with a characteristic hour-glass shape by E9.25 (Figure 1A–C and Tables 2 & 3), as previously described for *Tbx20* ^*-/-*^ hearts [3,13–15]. Embryos that were Tbx20^+/-^; Tbx3^-/-^, on the other hand, initiated heart looping normally but some displayed atrioventricular convergence defects by E9.25 (2/4) similar to Tbx3^-/-^ embryos (4/4) (Figure 1E–G and Table 3), as previously described [19]. Wild type and single or double heterozygotes had normal heart morphology (n=120).

**Table 2:** Frequency of different morphological defects in embryos at E8.5 from matings of *Tbx20* ^*+/-*^ *; Tbx3* ^*+/-*^ double heterozygotes^*^.

		Genotypes					
Stage	Morphology	wt**	*Tbx20* ^*-/-*^ *; Tbx3* ^*+/+*^	*Tbx20* ^*-/-*^ *; Tbx3* ^*+/-*^	Tbx20^+/+^ *;Tbx3* ^*-/-*^	*Tbx20* ^*+/-*^ *; Tbx3* ^*-/-*^	*Tbx20* ^*-/-*^ *; Tbx3* ^*-/-*^
E8.5	Normal	89	3***	2***	1***	0	1***
	Abnormal. looping	0	14	14	0	0	0
	Abnormal looping / delay	0	3	3	0	0	3
	Abnormal looping / dead	0	2	0	0	0	0
	Delay	6	1	6	7	4^##^	3
	Dead^#^	1	0	3	0	0	1
Total (n=167)		96	23	28	8	4	8

* Double heterozygotes were either intercrossed or crossed to single heterozygotes.

** wt, wild type; this group includes wild type and heterozygotes for one or both alleles.

*** Embryos were at the cardiac crescent stage, prior to heart looping.

# Approximate time of death between E8.0 and E8.5; too degenerate to score for heart phenotype.

## One of these embryos had a yolk sac defect.

**Table 3:** Frequency of different morphological defects in embryos at E9.25 from matings of *Tbx20* ^*+/-*^ *; Tbx3* ^*+/-*^ double heterozygotes*.

		Genotypes					
Stage	Morphology	wt**	*Tbx20* ^*-/-*^ *; Tbx3* ^*+/+*^	*Tbx20* ^*-/-*^ *; Tbx3* ^*+/-*^	Tbx20^+/+^ *;Tbx3* ^*-/-*^	*Tbx20* ^*+/-*^ *; Tbx3* ^*-/-*^	*Tbx20* ^*-/-*^ *; Tbx3* ^*-/-*^
E9.25	Normal	23	0	0	0	0	0
	Abnormal looping	0	0	0	0	0	1
	Abnormal looping / delay	0	1	1	0	0	4***
	Abnormal convergence	0	0	0	1	0	0
	Abnormal convergence /delay	0	0	0	2	1	0
	Abnormal convergence / dead	0	0	0	1	1	0
	Delay	0	0	0	0	1	0
	Dead^#^	1	3	0	0	1	1
Total (n=43)		24	4	1	4	4	6

* Double heterozygotes were either intercrossed or crossed to single heterozygotes.

** wt, wild type; this group includes wild type and heterozygotes for one or both alleles.

*** One of these embryos had a yolk sac defect.

# Approximate time of death between E8.75-E9.0; too degenerate to score for heart phenotype.

Limb abnormalities in *Tbx3* ^*-/-*^ embryos were not observed between E8.5 and E9.25, which is in agreement with previous work [18]. The only abnormality seen in yolks sac or gross placental morphology was a reduction in yolk sac vasculature in 1 of 4 *Tbx20* ^*+/-*^ *; Tbx3* ^*-/-*^ embryos at E8.5 and 1 of 6 double homozygous mutant embryos at E9.25 (Table 3). This resembled the reduced vasculature reported for *Tbx3* ^*-/-*^ and *Tbx20* ^*-/-*^ embryos at E9.5 [3,18].

### Developmental delay in *Tbx3^-/-^* and *Tbx20^-/-^; Tbx3^-/-^* embryos

**Figure 2:**
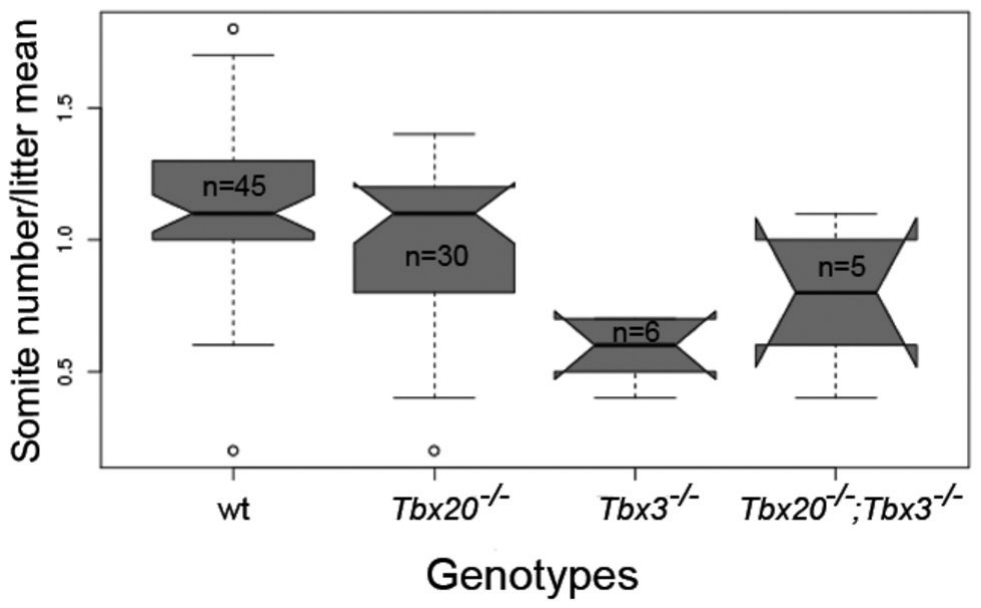
*Tbx20* ^*-/-*^ *; Tbx3* ^*-/-*^ double homozygotes are developmentally delayed as are *Tbx3* ^*-/-*^ embryos. Notched box plots showing the distributions of the ratio of somite number in an embryo / the mean somite number for the litter in four different groups: wild type, *Tbx20* ^*-/-*^ homozygous mutants*;* Tbx3^-/-^ homozygous mutants and double homozygotes. Compound and single mutants were combined for this comparison as single mutants were not different from the compounds (Mann Whitney U test). Whiskers of the notched box plots represent 10th and 90th percentiles, boxes include the 25th through 75th percentile, and outliers are individually plotted. If the notches of two plots do not overlap, this indicates that the medians differ between the two. With respect to developmental stage, *Tbx20* ^*-/-*^ *; Tbx3* ^*-/-*^ double homozygotes resemble *Tbx3* ^*-/-*^ embryos and both groups are delayed compared to wild type or *Tbx20* ^*-/-*^ embryos.

Among the embryos from *Tbx20* ^*+/-*^ *; Tbx3* ^*+/-*^ double heterozygous crosses at E8.5, some single or double homozygous embryos showed developmental delay, but over 90% were living (65/71) (Table 2); by E9.25, however, 37% (7/19) were dead (Table 3). Thus, to assess the extent of developmental delay associated with each genotype prior to death, we scored living embryos at E8.5 (n=160) using somite number to determine developmental stage. To account for between-litter variation, the ratio of the number of somites for an embryo to the mean number of somites in its litter was calculated for each embryo. A Mann Whitney U test showed that there is no significant difference in this ratio between *Tbx20* ^*-/-*^ homozygous mutants and *Tbx20* ^*-/-*^ *;* Tbx3^+/-^ compound mutant embryos, nor between *Tbx3* ^*-/-*^ homozygous mutants and *Tbx20* ^*+/-*^ *; Tbx3* ^*-/-*^ compound mutants. Thus, single and compound mutants were combined and the distributions of ratios for embryos of each group were compared using notched box plots (Figure 2). The analysis showed that both *Tbx20* ^*-/-*^ *;* Tbx3^-/-^ double homozygotes and Tbx3^-/-^ mutants are significantly delayed developmentally compared to wild type or Tbx20^-/-^ embryos, but are similar to one another. Thus, with respect to developmental delay, *Tbx20* ^*-/-*^ *;* Tbx3^-/-^ embryos resemble Tbx3^-/-^ embryos (Figure 2).

### Cardiac gene expression in *Tbx20* ^*-/-*^ *; Tbx3* ^*-/-*^ embryos

**Figure 3:**
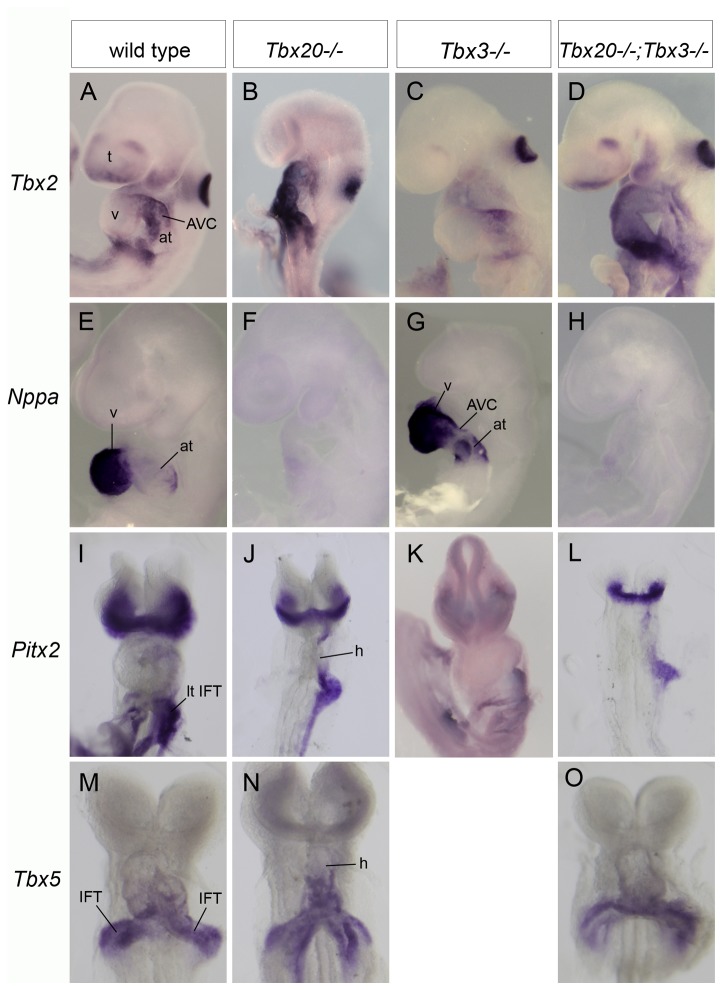
Cardiac marker gene expression suggests that *Tbx20* ^*-/-*^ *; Tbx3* ^*-/-*^ double homozygous mutants show a *Tbx20* ^*-/-*^ phenotype. Whole mount ISH left (A–H) or ventral (I–O) views of the head and heart of stage-matched wild type, single and double mutant embryos showing expression of *Tbx2* (E9.25; A-D), *Nppa* (E9.25; E-H)*, Pitx2* (E8.5; I-L) and *Tbx5* (E9.25; M-O). *Tbx2* is normally expressed in the AVC in wild type and *Tbx3* ^*-/-*^ hearts but is ectopically expressed in Tbx20^-/-^ and double homozygotes. In Tbx20^-/-^ and double homozygotes, *Nppa* expression is severely down regulated in ventricular myocardium, but in Tbx3^-/-^ hearts it is precociously upregulated in atrial myocardium. There is no change in *Pitx2* expression in the left inflow tract in Tbx20^-/-^, Tbx3^-/-^ or double homozygous embryos when compared to wild type. *Tbx5* is expressed in the atrioventricular and atrial progenitors in an antero-posterior gradient. There is no change in *Tbx5* expression in either Tbx20^-/-^ or double homozygotes compared to wild type at E9.25. at, atrium; AVC, atrioventricular canal; h, heart; IFT, inflow tract; lt IFT, left inflow tract; t, telencephalon; v, ventricle.

Molecular markers of cardiac development were used to further characterize the phenotype of compound and double homozygous mutant embryonic hearts. *Tbx2* is normally expressed in the non-chamber myocardium, most prominently in the AVC. As previously reported [14], *Tbx2* expression was not altered in *Tbx20* ^*-/-*^ hearts at E8.75 (data not shown) but was upregulated and ectopically expressed throughout the entire Tbx20^-/-^ mutant heart by E9.25. Similar upregulation and ectopic expression of *Tbx2* was seen in *Tbx20* ^*-/-*^ *; Tbx3* ^*-/-*^ double homozygous mutant hearts, whereas expression in *Tbx3* ^*-/-*^ hearts was normal (Figure 3A–D).

Expression of *Nppa*, a marker of chamber myocardium and a target of both Tbx20 and Tbx3, was severely down regulated in both *Tbx20* ^*-/-*^ and double homozygous mutant hearts from E8.5-E9.25, demonstrating the lack of chamber differentiation. In contrast, precocious expression of *Nppa* in atrial myocardium was observed in Tbx3^-/-^ embryos, as previously described [19] (Figure 3E–H), as well as in *Tbx20* ^*+/-*^ *; Tbx3* ^*-/-*^ compound mutants (data not shown).

We observed no change in expression of *Pitx2*, which is important for cardiac remodeling and asymmetric development, in the left inflow tract (IFT) of Tbx20^-/-^, Tbx3^-/-^ or Tbx20^-/-^ *;* Tbx3^-/-^ embryos compared to stage-matched wild type controls (Figure 3I–L). *Tbx5*, which is expressed in the atrioventricular and atrial progenitors in an antero-posterior gradient, was slightly reduced in both Tbx20^-/-^ and Tbx20^-/-^ *;* Tbx3^-/-^ embryos at E8.5 but no difference in expression was evident among the genotypes by E9.25 (Figure 3M–O).

## Discussion

The DNA binding domain of T-box proteins, as well as T-box binding motifs identified in the promoters of downstream target genes, are highly conserved. This leads to the possibility that these transcription factors have targets in common and could compete or cooperate in transcriptional regulation of specific targets in areas of overlapping gene expression. Furthermore, some T-box proteins can bind DNA as dimers, raising the possibility of heterodimerization. In the developing heart at least seven T-box genes are expressed [1,23] and although each has a specific pattern of expression, there are extensive areas of expression overlap. Genetic studies have shown that some of these genes do indeed interact during heart development. *Tbx1*, *Tbx2* and *Tbx3* all interact in OFT development with *Tbx1* upstream of *Tbx2* and *Tbx3*. Double homozygous mutants for *Tbx1* with either *Tbx2* or *Tbx3* have heart failure more severe than either single mutant alone [24]. At the level of gene regulation, *Tbx2* and *Tbx5* have been shown to repress or activate, respectively the expression of *Nppa* through competing interactions with the transcriptional co-factor Nkx2-5 [25–27]. *Tbx3* and *Tbx20* have also been shown capable of interacting with Nkx2-5 [12,17]. T-box genes might also regulate one another and in this respect, *Tbx20* has been shown to repress *Tbx2* expression in the developing heart, as in its absence, *Tbx2* is upregulated throughout the chamber myocardium of the heart tube [3].

*Tbx20* and *Tbx3* have areas of overlapping expression in the OFT and AVC of the developing heart and thus have the potential to interact either by affecting common downstream target genes or by cross regulation. A potential example of cross regulation was noted in *Tbx3* mutant hearts at E13.5-14.5 where *Tbx20*, which is normally expressed in the base of the developing interventricular septum, appears to be ectopically expressed in the developing conduction system at the apex of the septum where *Tbx3* would normally be expressed [10]. However, whether this is genuine ectopic expression or simply the lack of *Tbx3*-expressing tissue in the abnormally developing septum is not clear. On the other hand, *Tbx20* regulates *Tbx3* in the AVC as deletion of *Tbx20* specifically in the AVC leads to a downregulation of *Tbx3* expression at E9.5 [21].

To investigate the phenotypic consequences of possible interactions between the two genes at the earliest stages of heart development, we produced double mutants using mutant alleles that ablate function and compared the morphological and molecular phenotypes of the single, compound and double mutants. We found no evidence for a genetic interaction in double heterozygotes, which were viable and fertile, or in double homozygotes, which displayed an embryonic heart phenotype indistinguishable from *Tbx20* single homozygous mutants: morphologically the double homozygous mutant hearts displayed a vertically oriented hourglass shaped, two-chambered heart with no looping. Molecularly, *Tbx2* was upregulated throughout the heart tube and *Nppa* was severely down regulated in the double homozygous mutants, similar to *Tbx20* single mutants, whereas *Pitx2* and *Tbx5* were unchanged. This phenotype is indistinguishable from the *Tbx20* ^*-/-*^ phenotype where cardiac development is arrested and chamber differentiation does not occur although the anterior/posterior and left/right axes are established correctly. Because of this early arrest starting at E8.5, cardiac development never reached the stage where phenotypic features characteristic of the *Tbx3* mutant heart, such as convergence defects, increased AVC cell proliferation and ventricular septal defects, could be evaluated. Thus, *Tbx20* is epistatic to *Tbx3* with respect to the early heart phenotype and heart development is arrested at an earlier developmental stage than the *Tbx3* phenotype becomes manifest. To further investigate potential genetic interactions in the cardiac conduction system, conditional alleles that allow survival beyond the time of death of the homozygous mutants would be required. For this type of study, the recently developed *Tbx3* allelic series, which has been used to demonstrate exquisite dose sensitivity of the cardiac conduction system to levels of Tbx3 in embryos and adults could be combined with a conditional *Tbx20* allele to test for genetic interactions throughout development and adult life [28]. It has been previously reported that *Tbx3* homozygous mutant embryos are delayed in their overall development compared with littermates [18]. We observed a similar developmental delay in *Tbx20* ^*-/-*^ *; Tbx3* ^*-/-*^ double homozygous embryos that was not seen in *Tbx20* single mutants. Thus, developmental delay is associated with the *Tbx3* mutant genotype, even though *Tbx20* mutants have a more severe and earlier heart abnormality. It was previously postulated that vascular defects and apoptosis in the *Tbx3* mutant yolk sac were responsible for this developmental delay [18], even though the subsequent discovery of a heart phenotype put this interpretation into question [19]. Both *Tbx3* and *Tbx20* are expressed in the yolk sac, *Tbx20* in the mesoderm layer [3] and *Tbx3* in both endoderm and mesoderm [29], and vascular deficiency has been reported in both single mutants at E9.5 [3,18]. In this study of E8.5-9.25 embryos, the incidence of abnormal yolk sac vasculature was very low in *Tbx3* mutants and was not exacerbated in double mutants. Thus the developmental delay associated with the *Tbx3* mutant genotype does not appear to be associated with an early yolk sac vasculature phenotype and remains to be elucidated.
